# Aging and the human gut microbiota—from correlation to causality

**DOI:** 10.3389/fmicb.2014.00764

**Published:** 2015-01-12

**Authors:** Sitaraman Saraswati, Ramakrishnan Sitaraman

**Affiliations:** ^1^Department of Biochemistry, Dayananda Sagar College of Arts, Science, and CommerceBangalore, India; ^2^Department of Biotechnology, TERI UniversityNew Delhi, India

**Keywords:** gut microbiota, microbiome, ecological succession, co-evolution, inter-species/kingdom signaling, elderly, aging, effector molecules

## Introduction

The human gastrointestinal (GI) tract harbors the largest number and concentration of microbes found in the human body. Perturbations in the gut microbial ecosystem have also been associated with conditions as diverse as chronic GI diseases (e.g., Crohn's disease, ulcerative colitis), metabolic disorders (e.g., diabetes types 1 and 2, obesity) and antibiotic use (for a review see Sekirov et al., 2010). Metagenomic culture-independent methods have enabled the unraveling of the complexity of the gut microbiota (Rajilić-Stojanović et al., 2009). Given the considerable inter-individual diversity in the actual composition of the microbiota, significant collaborative attempts have been made to systematize the available knowledge (Arumugam et al., 2011; Human Microbiome Project Consortium, 2012) and identify “core” microbiota that are conserved among humans to facilitate meaningful comparisons (Huse et al., 2012). Changes in the microbial composition also take place with age, with a high degree of variability at the two extremes of infancy and old age, punctuated by comparative stability during adulthood (for reviews, see Woodmansey, 2007; O'Toole and Claesson, 2010). Given that increases in life expectancy will likely result in an increase in the elderly population worldwide, analysis of the contribution of the microbiota to healthy aging assumes greater significance (for a recent review, see Tiihonen et al., 2010).

Age-related spatio-temporal variations in the microbiota are best viewed within an ecological- evolutionary framework (see review by Costello et al., 2012). Diet is a major, controllable environmental factor influencing the composition of the host microbiome, with the high-fat, sugar-rich Western diet contributing to a *Bacteroides*-dominant microbiome and high-fiber diet to one dominated by Firmicutes with a strong correlation between long-term diet and enterotypes (Wu et al., 2011). In terms of ecological succession, the Bifidobacterium-dominated microbiota of the infant changes over time into the Bacteroidetes- and Firmicutes-dominated microbiota of the adult (Ottman et al., 2012), remaining fairly stable through adulthood in the absence of perturbations like long-term dietary changes or repeated antibiotic intervention. Pathogens may then be viewed as invasive species in the ecological sense, constantly testing the resilience of the native ecosystem, resulting in their elimination, low-level persistence (enabling future opportunism), or establishment causing disease.

## Age- and environment-related changes in the gut microbiota

The most noticeable feature in the microbiota of elderly individuals is an alteration in the relative proportions of the Firmicutes and the Bacteroidetes, with the elderly having a higher proportion of Bacteroidetes while young adults have higher proportions of Firmicutes (Mariat et al., 2009). Significant decreases in Bifidobacteria, *Bacteriodes*, and *Clostridium* cluster IV have also been reported (Zwielehner et al., 2009). Variability among individuals ranges from 3% to 92% for Bacteroidetes and between 7% and 94% for Firmicutes. The microbiota of individual subjects however exhibit less temporal variability (Claesson et al., 2011).

Changes occurring in the microbiota during aging can have an impact on host health. van Tongeren et al. (2005) studied the relationship between microbial diversity and frailty scores in elderly individuals. A significant reduction in the proportion of lactobacilli, *Bacteroides/Prevotella* and *Faecalibacterium prausnitzii*, and an increase in the proportion of *Ruminococcus*, *Atopobium*, and Enterobacteriaceae was seen in individuals with high frailty scores. Recently, Claesson et al. (2012) studied the relationship between diet, host health, environment, and the gut microbiota. Specifically, association was observed between microbial diversity and the functional independence measure (FIM), the Barthel index (used to evaluate performance in daily routine activities) and nutrition. Decreased microbial diversity correlated with increased frailty, decreased diet diversity and health parameters, and with increased levels of inflammatory markers. Individuals living in a community had the most diverse microbiota and were healthier as compared to those in short- or long-term residential care. Bartosch et al. (2004) reported that hospitalization itself appeared to result in a decreased abundance of the *Bacteroides-Prevotella* group. Later studies by Claesson et al. (2012) further detailed the effects of residence location on gut microbial diversity. Residence location also affects the microbiota of patients on antibiotic treatment, with highest levels of bifidobacteria in the community-dwelling group and lowest in those in long-term residential care. The levels of *Lactobacillus* in the antibiotic-untreated group were higher in rehabilitation (hospital stay < 6 weeks) as compared to long-stay or community-dwellers.

Predictably, antibiotic treatment has been reported to affect both richness and diversity of the microbiota and is associated with decreases in bifidobacteria (Bartosch et al., 2004; Woodmansey et al., 2004; O'Sullivan et al., 2013) as well as the *Bacteroides-Prevotella* group (Bartosch et al., 2004; Woodmansey et al., 2004). Lactobacilli, however, are observed to have increased in antibiotic-treated elderly subjects (Woodmansey et al., 2004; O'Sullivan et al., 2013); similarly, an increase in clostridial diversity has also been reported (Woodmansey et al., 2004). The changes taking place in response to antibiotics are more apparent at the genus rather than the family or phylum levels (O'Sullivan et al., 2013). Treatment with antibiotics can result in *Clostridium difficile* infection in the elderly, manifesting as *C. difficile*-associated diarrhea (CDAD). Reduced species diversity in CDAD patients compared to healthy elderly and young adults accompanied by a large reduction in bifidobacteria, *Bacteroides*, and *Prevotella* has been reported (Hopkins and MacFarlane, 2002). However, an increase in facultative bacteria along with an increase in diversity of clostridial and lactobacilli species in CDAD patients was reported in the same study. A recent study also detected differences at the genus level between *C. difficile* -negative and -positive subjects, and patients with CDAD (Rea et al., 2012). Incidentally, the isolation of the hypervirulent *C. difficile* R027 ribotype from one asymptomatic individual in this study who exhibited greater microbial diversity compared to CDAD patients, serves to highlight the importance of an intact and unperturbed gut ecosystem in resisting colonization by pathogens. Restoration of the microbiota and curing CDAD by fecal microbiota transplantation (FMT) in recent years presents a novel therapeutic strategy that is under intense scrutiny (for a discussion see Vrieze et al., 2013). In contrast to antibiotics, the common usage of non-steroidal anti-inflammatory drugs (NSAIDs) does not appear to significantly perturb the microbiota (Tiihonen et al., 2008; Mäkivuokko et al., 2010).

Interestingly, centenarians harbor less diverse microbiota, though Bacteroidetes and Firmicutes still constitute the dominant phyla (Biagi et al., 2010), with enrichment for potentially pathogenic Proteobacteria. Biagi et al. (2010) reported higher levels of *Akkermansia* in the elderly, compared to young adults, in contrast to an earlier study (Collado et al., 2007) that reported a decrease in this genus with age. Subsequent functional microbiome profiling of selected, well-characterized samples from this cohort indicated increased abundance of genes involved in aromatic amino acid metabolism, decreased abundance of those involved in short-chain (≤6) fatty acid production and an enrichment of “pathobionts”—low-abundance microbiota that promote and sustain pro-inflammatory conditions (Rampelli et al., 2013). This supports an earlier finding by Collino et al. (2013) that increased levels of phenylacetylglutamine (PAG) and *p*-cresol-sulfate (PCS), derived from the catabolism of aromatic amino acids, were excreted in the urine of centenarians. Thus, the changes in the gut microbiota of the elderly are reflected in the changes in the microbial metabolism.

## From correlation to causality—some general considerations

High-throughput analytical tools and meta-“omics” enable probing of the host-microbiota relationship at high resolution, helping correlate healthy or diseased states with the detailed composition of the microbiota, and informing the use of well-characterized (e.g., probiotic) or largely unknown (e.g., stool transplants) mixtures of microorganisms for restorative or maintenance purposes. However, complicating matters further is the existence of distinct ecological niches all along the alimentary canal, indicating that the common (and convenient) method of fecal sampling for microbiota studies may not adequately reflect the situation *in vivo* (Li et al., 2011). Ideally, we would like to determine the identity of the molecules that mediate host-microbiota interactions, and how their deployment is regulated. Here, information about host-pathogen interactions and general microbiology offers insights into the range of intra- and inter-species interactions, and even inter-kingdom ones (Table 1). However, given our current inability to convincingly delineate the contextually most significant effector mechanisms involved in the host-microbiota interaction over a lifetime, it is difficult to tease apart causality from correlation. Moreover, the host and the microbiota impact each other reciprocally, and the microbiota themselves interact in many modes among themselves. While current host signals may modulate the microbiota, it is an open question whether these signals themselves were induced, at least in some measure by components of the microbiota themselves. Theoretically, the host could also modulate the microbiota so that microbial responses are, in turn, beneficial to itself. The landmark study of Claesson et al. (2012) points to the possibility of such a reciprocal (and more confusingly, recursive) relationship between host health and microbial diversity.

**Table 1:** **Examples of effector mechanisms involved in inter-species and inter-kingdom interactions**.

**Effector molecule(s)**	**Relevant aspects for microbiota studies**	**Reference(s)**
Secreted phosopholipases (PldA and PldB) of *Pseudomonas aeruginosa*.	Killing of competing bacteria (PldA, PldB), promoting *P. aeruginosa* internalization by host cells (PldB).	Russell et al., 2013; Jiang et al., 2014
Autoinducer-2 (AI-2)	Mediates inter-species signaling among the microbiota. Production of AI-2 by *Bifidobacterium breve* UCC2003 (a probiotic strain) is required for gut colonization and protection against *Salmonella* infection in *C. elegans.*	Christiaen et al., 2014
Autoinducer-3 (AI-3), epinephrine and norepinephrine	Autoinducer 3 (AI-3) produced by the intestinal microbiota can cross-signal with host hormones epinephrine and norepinephrine.	Sperandio et al., 2003
	Epinephrine/Norepinephrine can be sensed by enterohemorrhagic *E. coli*, inducing the expression of the LEE pathogenicity island and the flagella regulon. The AI-3 signaling cascade is conserved across several bacterial species.	Reading and Sperandio, 2006

From an evolutionary standpoint, we would also like to know how much these interactions and associations are modulated over the host lifetime and during co-evolution in order to benefit both partners. Their persistence is also dependent on the forces of selection operative at a given time (Sancar, 2008; Lukeš et al., 2011), such as bacteriophage infection (Reyes et al., 2012; Koskella, 2013). The recent discovery that an unknown secreted protein from human intestinal cells decreases conjugation efficiency in *E. coli* indicates that the host can potentially influence the composition, the rate of evolution and lateral gene transfer among its microbiota (Machado and Sommer, 2014). An unexplored consideration is the potential influence of host hormones and their changing levels over age on the microbiota. Additionally, the relative abundance of a given enterotype or species may also not be an unambiguous predictor of relative importance in the ecological sense. Therefore, identifying keystone species that might have an effect on the ecosystem disproportionate to their abundance would be very valuable for focused studies of the microbiota. We surmise that the benefits arising from probiotic administration could be due to their temporary assumption of such a “keystone” role. Notably, current studies of microbiota concentrate solely on the doubtlessly important bacteria, omitting archaea and clinically relevant eukaryotes (fungi), an approach that could potentially miss less abundant but nevertheless important species. A recent finding on the important role of Dectin-1, a C-type lectin receptor and an innate immune sensor of fungi, in preventing intestinal colitis-associated pathology underlines the importance of interactions between the human host and the numerically less abundant intestinal fungi (Iliev et al., 2012).

Natural selection operates simultaneously at multiple ecological levels, ranging from single unicellular organisms to entire communities and ecosystems. The magnitude and relative importance of multiple, and often stochastic, selection pressures acting over a human lifetime, therefore need careful consideration. Thus, it could be inaccurate to ascribe a given microbiota profile solely to a single factor (e.g., diet) even though there may be some correlation between the said profile and a single contributing factor (see review by Yeoman et al., 2011). Additionally, non-equilibrium (co-) evolutionary processes may not necessarily result in optimality. Rather, they are governed by the actual functionality of the given arrangement (“phenotype”) and its ability to propagate itself (fitness), not on the details of the arrangement itself (components, genotypes etc.). Microbiota research, in the context of aging or otherwise, will greatly benefit from the integration of several disparate pieces of mechanistic information within an evolutionary-ecological framework in order to determine the causes underlying our observations, and the formulation of plausible mechanistic models describing how these causes result in the observed effects.

### Conflict of interest statement

The authors declare that the research was conducted in the absence of any commercial or financial relationships that could be construed as a potential conflict of interest.
